# Staged visceral revascularization prior to central aortic repair in acute type A aortic dissection complicated by mesenteric malperfusion: a case report

**DOI:** 10.1093/ehjcr/ytag693

**Published:** 2026-09-19

**Authors:** Georges Abi Abdallah, Francesca Pitocco, Antoine Cazelles, Marie Corniquet, Charles Henri Gautier, Lena Ple-Nemo, Maxime Renaux, Nicolas Polge

**Affiliations:** Department of Anaesthesiology, Critical Care & Perioperative Medicine, AP-HP, Hôpital Européen Georges Pompidou, 20 rue Leblanc, 75015 Paris, France; Université Paris Cité, Inserm, PARCC, UMR_S970, 56 rue Leblanc, 75015 Paris, France; Université Paris Cité, Inserm, PARCC, UMR_S970, 56 rue Leblanc, 75015 Paris, France; Department of Radiology, Hôpital Européen Georges Pompidou, Assistance Publique Hôpitaux de Paris, 20 rue Leblanc, 75015 Paris, France; Department of Digestive and Oncological Surgery, Hôpital Européen Georges Pompidou, 20 rue Leblanc, 75015 Paris, France; Department of Vascular Surgery, AP-HP, Hôpital Européen Georges Pompidou, 20 rue Leblanc, 75015 Paris, France; Department of Cardiac Surgery, AP-HP, Hôpital Européen Georges Pompidou, 20 rue Leblanc, 75015 Paris, France; Department of Anaesthesiology, Critical Care & Perioperative Medicine, AP-HP, Hôpital Européen Georges Pompidou, 20 rue Leblanc, 75015 Paris, France; Department of Anaesthesiology, Critical Care & Perioperative Medicine, AP-HP, Hôpital Européen Georges Pompidou, 20 rue Leblanc, 75015 Paris, France; Department of Anaesthesiology, Critical Care & Perioperative Medicine, AP-HP, Hôpital Européen Georges Pompidou, 20 rue Leblanc, 75015 Paris, France

**Keywords:** Acute type A aortic dissection, Mesenteric malperfusion, Visceral ischaemia, Multidisciplinary care, Aortic surgery, Visceral revascularization, Case report

## Abstract

**Background:**

Acute type A aortic dissection (ATAAD) complicated by mesenteric malperfusion is among the most lethal cardiovascular emergencies, combining the immediate threat of aortic rupture with rapidly progressive visceral ischaemia. The optimal sequence of intervention remains controversial. Although current guidelines recommend immediate central aortic repair, this strategy may carry particularly high perioperative risk when advanced mesenteric ischaemia is already present.

**Case summary:**

A 54-year-old woman with no relevant medical history presented with ATAAD extending from the sinotubular junction to the iliac bifurcation, complicated by severe mesenteric malperfusion and haemodynamic instability. Following urgent multidisciplinary discussion, a staged strategy was adopted. On Day 0, visceral reperfusion was achieved by iliac-to-superior mesenteric artery bypass with concomitant exploratory laparotomy, combined with thoracic endovascular aortic repair. Progressive intestinal ischaemia over the next 48 h required multiple abdominal surgeries, including right colectomy, left colectomy with ileosigmoidostomy, and cholecystectomy, until bowel viability was controlled. Definitive total aortic arch replacement using a Thoraflex hybrid prosthesis was then performed on Day 6 under cardiopulmonary bypass with brief circulatory arrest. Despite a prolonged and complex postoperative course, the patient survived to hospital discharge.

**Discussion:**

This case illustrates the multidisciplinary decision-making involved in the management of a highly selected patient with ATAAD and established severe mesenteric malperfusion syndrome, in whom the immediate risk of visceral ischaemia was weighed against the substantial risk of delaying central repair.

Learning Points

- Emergency central repair remains the standard treatment for acute type A aortic dissection with mesenteric malperfusion.
- A visceral-first strategy may be considered in highly selected patients with severe mesenteric malperfusion syndrome.
- Early visceral reperfusion may limit irreversible bowel injury and create a safer window for definitive aortic repair.
- If central repair is deferred, close multidisciplinary surveillance and early definitive repair are essential.

## Introduction

Acute Type A aortic dissection (ATAAD) is a life-threatening surgical emergency associated with persistently high mortality despite continuous improvements in surgical techniques and perioperative care.^1,2^ Given the imminent risks of aortic rupture, cardiac tamponade, and stroke, emergency central aortic repair is universally recommended as the definitive treatment by current international guidelines.^3–5^

However, this paradigm is challenged when ATAAD is complicated by malperfusion syndrome, particularly mesenteric malperfusion, which carries mortality approaching 50% despite surgical intervention.^6–8^ When visceral perfusion is compromised by dynamic or static obstruction of the mesenteric vessels, bowel ischaemia may progress rapidly, with rising lactate levels and impending multiorgan failure. In such circumstances, proceeding directly to cardiopulmonary bypass may exacerbate systemic injury, delay the surgical source control of ischaemia, and further reduce the likelihood of survival.^9^

The optimal therapeutic sequence in this setting remains highly controversial. Current ESC, EACTS/STS, and ACC/AHA guidelines recommend immediate central aortic repair as the standard strategy but also recognize that this approach may be insufficient when visceral ischaemia is already established. In carefully selected patients, upfront visceral revascularization before definitive aortic repair may therefore be considered as a temporizing strategy, although the supporting evidence remains limited.^3–5^

We report a case of ATAAD with severe mesenteric malperfusion in which a multidisciplinary team elected to perform visceral revascularization first, followed by staged abdominal surgery and delayed aortic arch repair. This case illustrates the multidisciplinary decision-making involved in a highly selected patient managed with a visceral-first strategy and underscores the need to balance competing surgical priorities when severe mesenteric malperfusion complicates ATAAD.

## Summary figure

**Figure:**
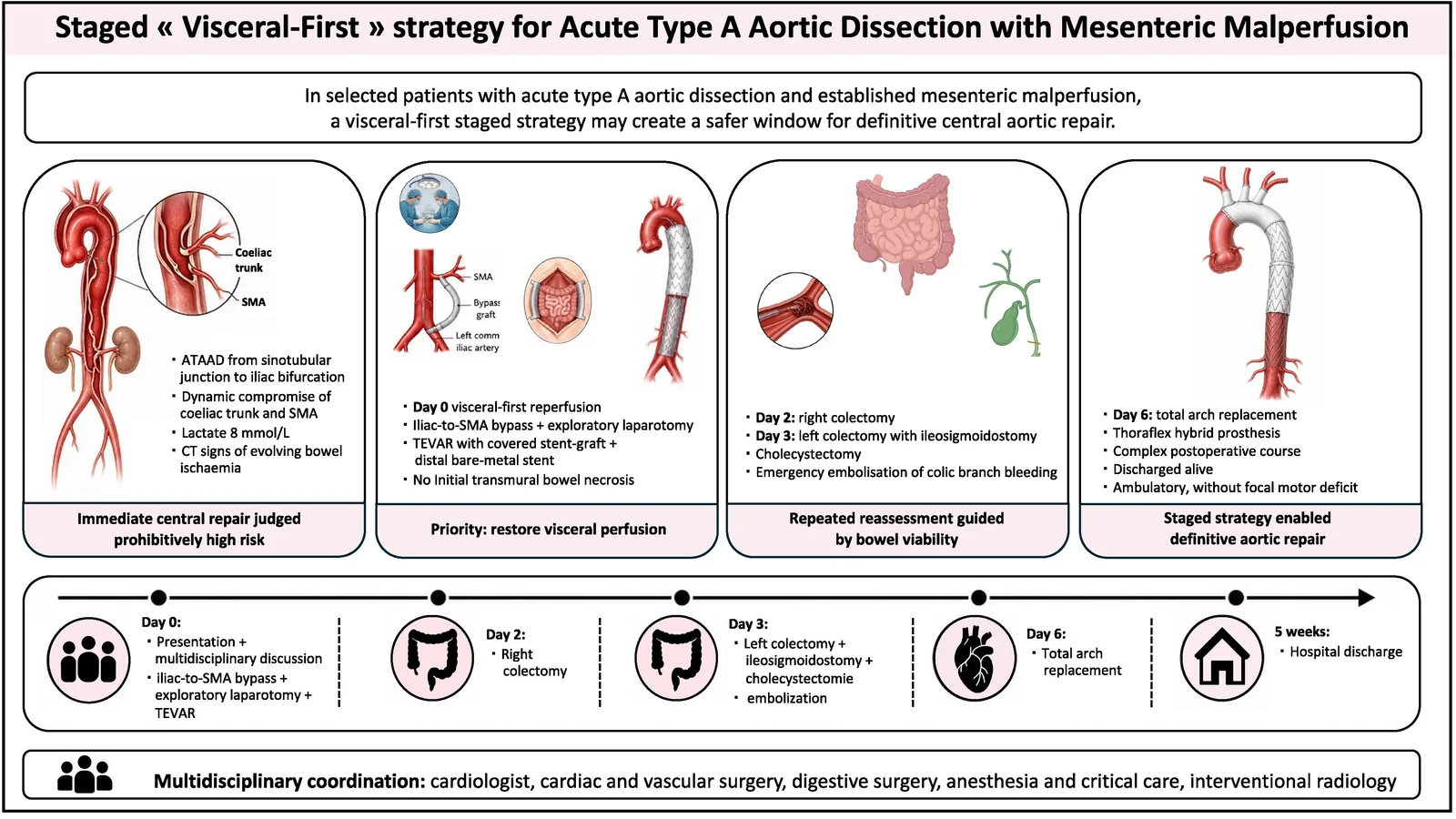


## Case presentation

A 54-year-old woman with no prior medical history presented with sudden-onset chest and abdominal pain radiating to both lower limbs, followed by a transient collapse. On initial assessment at a nearby emergency department, blood pressure was 240/110 mmHg, symmetric in both arms, with sinus rhythm at 80–90 bpm. Neurological exam was normal (Glasgow Coma Scale 15), with no respiratory distress on room air, preserved lower limb pulses, and no evidence of peripheral ischaemia.

Emergency computed tomography angiography demonstrated an acute type A aortic dissection originating at the sinotubular junction and extending to the iliac bifurcation, including both external iliac arteries (*Figure 1A*). The supra-aortic vessels arose from the false lumen, with ∼50% stenosis of the left common carotid artery over 5–6 cm and extension of the dissection flap to the origin of the brachiocephalic trunk. At the abdominal level, the coeliac trunk and superior mesenteric artery (SMA) came from the true lumen but were dynamically compromised by compression from the false lumen (*Figure 1B*). These findings were associated with circumferential thickening of the right colonic wall and a suspected mural defect of the transverse colon, consistent with early intestinal ischaemia (*Figure 1C* and *D*). The left renal artery arose from the false lumen, with preserved renal perfusion, whereas the right renal artery was dissected at its origin, with associated upper- and mid-polar renal infarction (*Figure 1B*). There was no imaging evidence of pericardial effusion or tamponade, significant aortic regurgitation, or coronary ostial involvement. Initial management included intravenous morphine and antihypertensive therapy, followed by transfer to our tertiary referral cardiac surgery centre. On arrival, the serum lactate level was 8.2 mmol/L (normal range 0.5–1.6 mmol/L), further supporting the diagnosis of severe mesenteric ischaemia.

**Figure 1:**
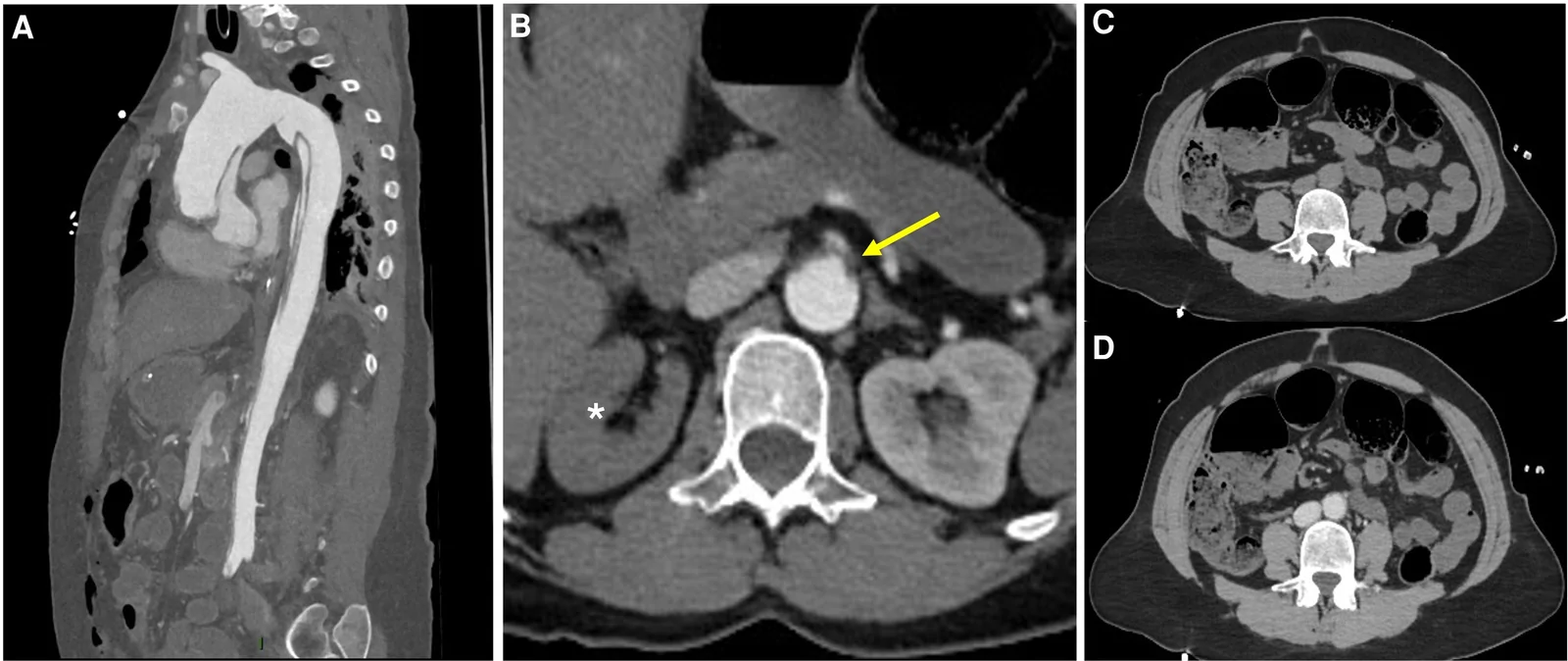
Computed tomography angiography at presentation demonstrating acute type A aortic dissection. (*A*) Sagittal view showing extension of the dissection from the sinotubular junction to the right common iliac artery. (*B*) Axial view demonstrating dynamic stenosis of the superior mesenteric artery due to compression by the false lumen (arrow), with associated right renal ischaemia (*). (*C*, *D*) Abdominal views in arterial and venous phases showing right colonic wall thickening and a suspected mural defect involving the right and transverse colon.

Given the severity of the mesenteric malperfusion syndrome, a multidisciplinary team involving cardiac, vascular, and digestive surgeons, anaesthesiologists, and critical care specialists considered immediate central aortic repair under cardiopulmonary bypass to carry a particularly high risk in this specific setting. Although the patient was initially hypertensive and controlled with continuous intravenous urapidil, she rapidly developed haemodynamic instability immediately before surgery, requiring discontinuation of antihypertensive therapy and norepinephrine infusion up to 0.45 µg/kg/min. In the context of established visceral ischaemia without an immediate proximal aortic complication, the team considered that restoring visceral perfusion and assessing bowel viability before definitive central repair offered the most appropriate risk-benefit balance. The factors informing this individualized decision are summarized in *Table 1*.

**Table 1:** Clinical, biological, imaging, and organizational factors informing the multidisciplinary decision to prioritize visceral reperfusion

	Factors supporting initial visceral reperfusion	Factors allowing consideration of temporarily deferred central repair
Clinical	Rapid haemodynamic deterioration requiring norepinephrine support	No clinical evidence of an immediately life-threatening proximal aortic complication
Biological	Marked hyperlactataemia (8.2 mmol/L, normal range 0.5–1.6)	—
Imaging	CT evidence of evolving bowel ischaemia; dynamic compression of the coeliac trunk and SMA by the false lumen	No pericardial effusion or cardiac tamponade; no significant aortic regurgitation; no coronary involvement; no evidence of aortic rupture
Organizational	Multidisciplinary assessment involving cardiac, vascular, digestive, anaesthesia, and critical care teams	Close multidisciplinary surveillance with immediate access to central aortic repair

CT, computed tomography; SMA, superior mesenteric artery.

Given the need for exploratory laparotomy to assess bowel viability, surgical mesenteric revascularization was favoured over isolated SMA stenting, which was considered technically less suitable because of the dissection anatomy. On Day 0, an iliac-to-SMA prosthetic bypass from the left common iliac artery was performed to provide direct mesenteric reperfusion (*Figure 2*), together with exploratory laparotomy and thoracic endovascular aortic repair (TEVAR) to address the broader dynamic component of malperfusion. TEVAR was performed through ultrasound-guided right common femoral access, with transoesophageal echocardiographic confirmation of true-lumen guidewire positioning. A 34/200-mm Gore CTAG covered stent-graft was deployed just distal to the left subclavian artery, followed by a 36/180-mm Cook Dissection bare-metal stent extending into the thoraco-abdominal aorta. Given the unrepaired proximal dissection, stent-graft oversizing and aggressive balloon expansion were avoided. Completion arteriography confirmed true-lumen expansion and adequate visceral perfusion. Exploratory laparotomy showed no definite transmural bowel necrosis (*Figure 3A*, Supplementary material online, *Video S1*), and planned reassessment was scheduled for the following day.

**Figure 2:**
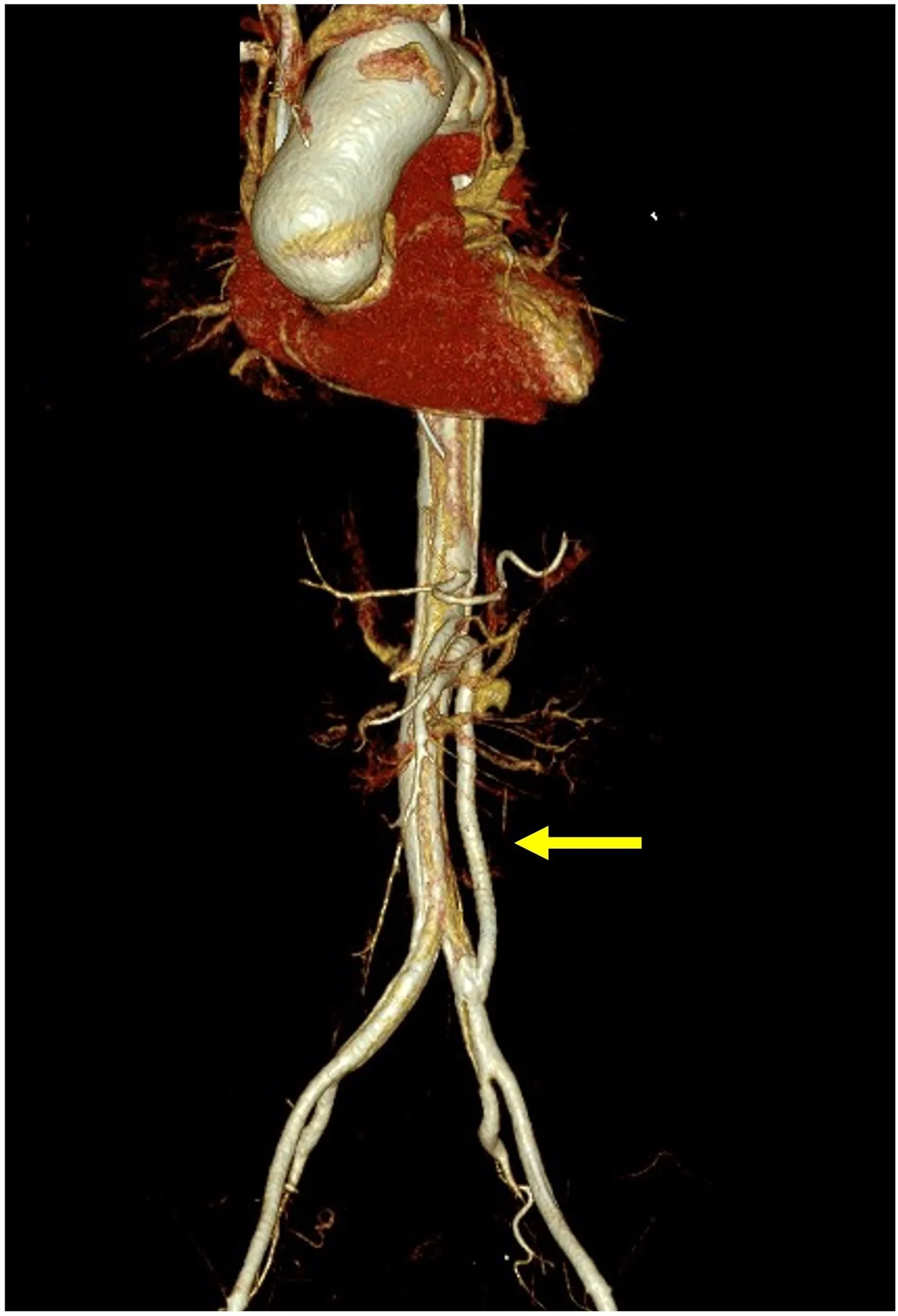
Three-dimensional computed tomography image obtained 1 day after surgery showing a patent iliac-to-superior mesenteric artery prosthetic bypass, with preserved blood flow through the coeliac artery and superior mesenteric artery.

**Figure 3:**
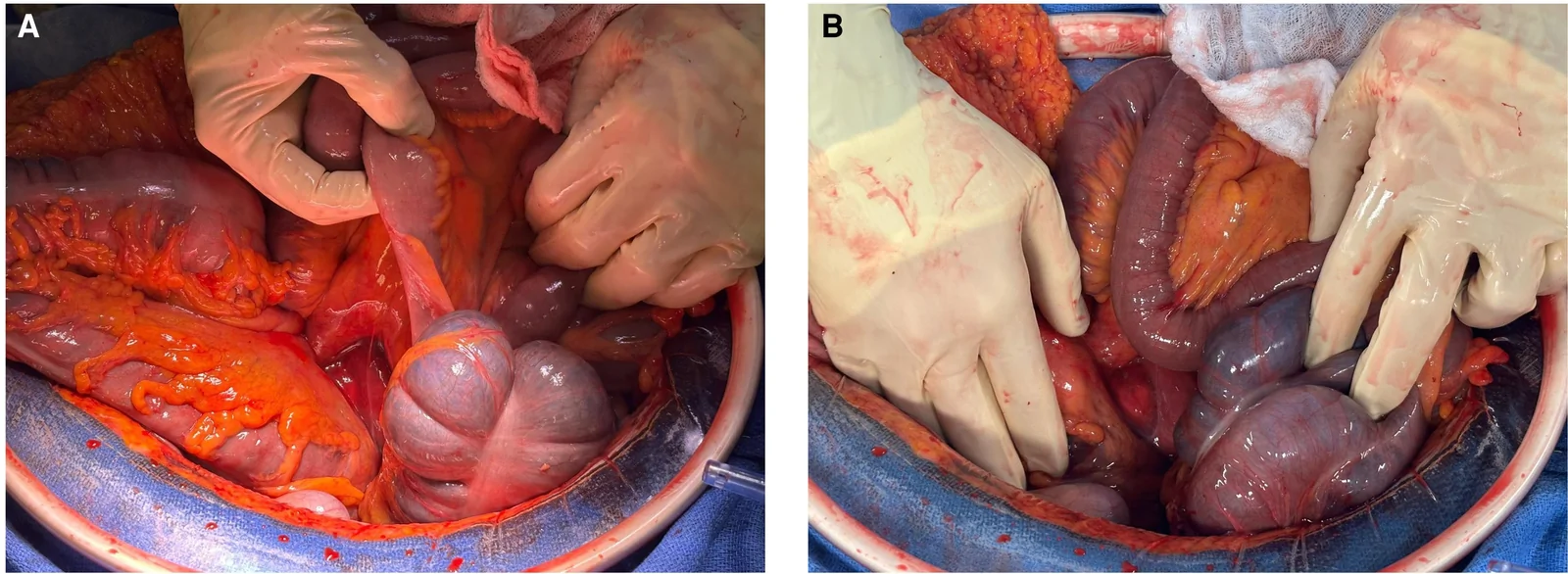
Intraoperative assessment of bowel viability during sequential exploratory laparotomies. (*A*) Initial exploratory laparotomy on Day 0 showing globally reassuring bowel appearance, without definite transmural necrosis. (*B*) Repeat exploratory laparotomy on Day 2 showing progression of colonic ischaemic injury, with marked caecal atony and worsening ischaemic changes, prompting right colectomy.

Postoperatively, severe metabolic acidosis persisted [pH 7.19 (normal range 7.35–7.45), lactate 9.3 mmol/L, base excess −12 mmol/L (normal range −2 to +2 mmol/L)], and the patient developed vasoplegic shock consistent with ischaemia-reperfusion syndrome, requiring norepinephrine infusion, aggressive haemostatic resuscitation, and invasive mechanical ventilation in the intensive care unit. Persistent haemodynamic instability on Day 2 prompted a second exploratory laparotomy. Compared with the previous assessment, the caecum was more atonic, and petechial lesions had developed in the transverse colon (*Figure 3B*). A right colectomy including the caecum and part of the transverse colon was therefore performed, with stapled closure of both ends pending delayed restoration of bowel continuity. Same-day rectosigmoidoscopy confirmed preserved viability of the rectum and left colonic mucosa up to the splenic flexure.

On Day 3, a third laparotomy revealed ischaemic necrosis of the splenic flexure together with gallbladder ischaemia. A left colectomy with ileosigmoidostomy and cholecystectomy were performed. Shortly afterwards, haemodynamic deterioration associated with active bleeding from the abdominal drains prompted emergency CT angiography, which identified a retroperitoneal haematoma with active extravasation from a branch of the transverse colic artery. Superselective catheterization through the struts of the bare-metal stent allowed successful glue embolization of the bleeding vessel (*Figure 4*). Following massive transfusion support, haemodynamic status improved, allowing progressive weaning of vasopressors.

**Figure 4:**
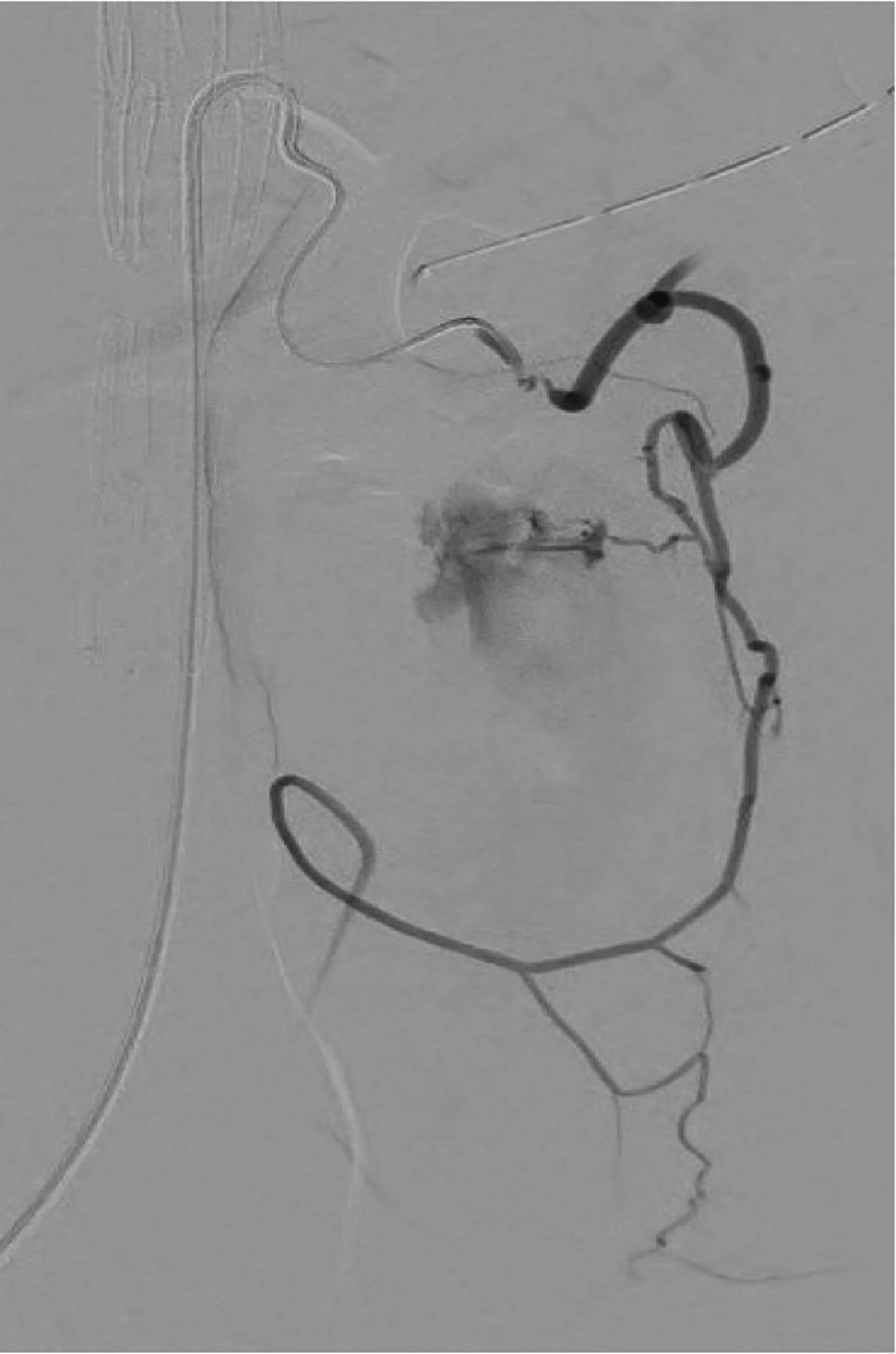
Emergency angiography and embolization of postoperative mesenteric bleeding. Catheterization was performed through the struts of the uncovered thoraco-abdominal endoprosthesis to access the superior mesenteric artery. Selective angiography demonstrated active extravasation from a branch of the middle colic artery. Because marked vessel tortuosity and vasospasm precluded further distal catheter advancement, embolization with diluted glue was performed, achieving successful haemostasis.

Throughout the interval before definitive aortic repair, the patient remained intubated in the intensive care unit under continuous invasive haemodynamic monitoring. During the initial postoperative period and sequential abdominal management, persistent circulatory shock required norepinephrine up to 1.2 µg/kg/min and vasopressin, precluding intravenous antihypertensive therapy. Following haemodynamic improvement and vasopressor withdrawal on Day 4, intravenous urapidil and labetalol were introduced, targeting a systolic blood pressure <120 mmHg and a heart rate of ∼60 bpm. Transthoracic echocardiography, performed at least twice daily, showed no new pericardial effusion, significant aortic regurgitation, or ventricular dysfunction suggestive of coronary involvement. Neurological monitoring included repeated pupillary examinations, continuous Bispectral Index monitoring, and bilateral frontal near-infrared spectroscopy (NIRS). No significant changes were observed during this period, and cerebral NIRS values remained symmetrical. Three CT examinations obtained during this interval for other clinical indications also allowed repeated assessment of the aorta, without evidence of proximal progression or impending rupture. Any evidence of a proximal aortic complication would have prompted immediate central repair. During the interval before definitive aortic repair, pharmacological thromboprophylaxis was provided with continuous intravenous unfractionated heparin because of severe acute kidney injury requiring renal replacement therapy and the need for repeated surgical procedures. Heparin was interrupted ∼6 h before each reintervention, with intermittent pneumatic compression used during interruption periods. Aspirin was introduced following prosthetic vascular implantation.

After haemostatic stabilization and sequential control of visceral ischaemia, definitive aortic repair was undertaken on Day 6 through median sternotomy. Cardiopulmonary bypass was established via right axillary artery and right atrial cannulation. Under moderate hypothermia (30°C) and antegrade cerebral perfusion, the aortic root was reconstructed with resuspension of the valve commissures, followed by supracoronary ascending aortic replacement and total arch replacement using a 36/100-mm Thoraflex hybrid prosthesis, selected to ensure an adequate distal interface with the previously implanted thoracic endograft. The supra-aortic vessels were separately reconstructed, with the left common carotid and left subclavian arteries connected through a trifurcated prosthetic graft and the brachiocephalic trunk reimplanted separately. Circulatory arrest was limited to 4 min. Myocardial protection was achieved with intermittent antegrade isothermic blood cardioplegia. Cardiopulmonary bypass and aortic cross-clamp times were 280 and 110 min, respectively. Postoperative transoesophageal echocardiography showed only minimal aortic regurgitation with preserved biventricular function. Perioperative haemostatic management was guided by an institutional transfusion algorithm based on point-of-care coagulation testing. Haemostatic support was substantial, requiring transfusion of 14 units of packed red blood cells, six units of fresh frozen plasma, eight platelet units, and 4 g of fibrinogen.

The postoperative course was prolonged due to ventilator weaning difficulties but ultimately favourable. Vasopressor support was weaned within 24 h, enteral nutrition was resumed on Day 7, and oliguric acute kidney injury requiring continuous venovenous haemofiltration resolved by Day 12. After initial extubation on Day 11, the patient developed acute neurological deterioration on Day 13, requiring reintubation. Brain imaging demonstrated a left cerebellar ischaemic stroke without haemorrhagic transformation. Concomitant imaging performed to assess the surgical repair demonstrated incomplete deployment of the distal Thoraflex segment with associated intraluminal thrombus formation, for which balloon dilatation was subsequently performed. Neurological improvement after sedation withdrawal allowed re-extubation, and follow-up brain magnetic resonance imaging confirmed left cerebellar and left posterior parietal ischaemic sequelae without new lesions (*Figure 5*).

**Figure 5:**
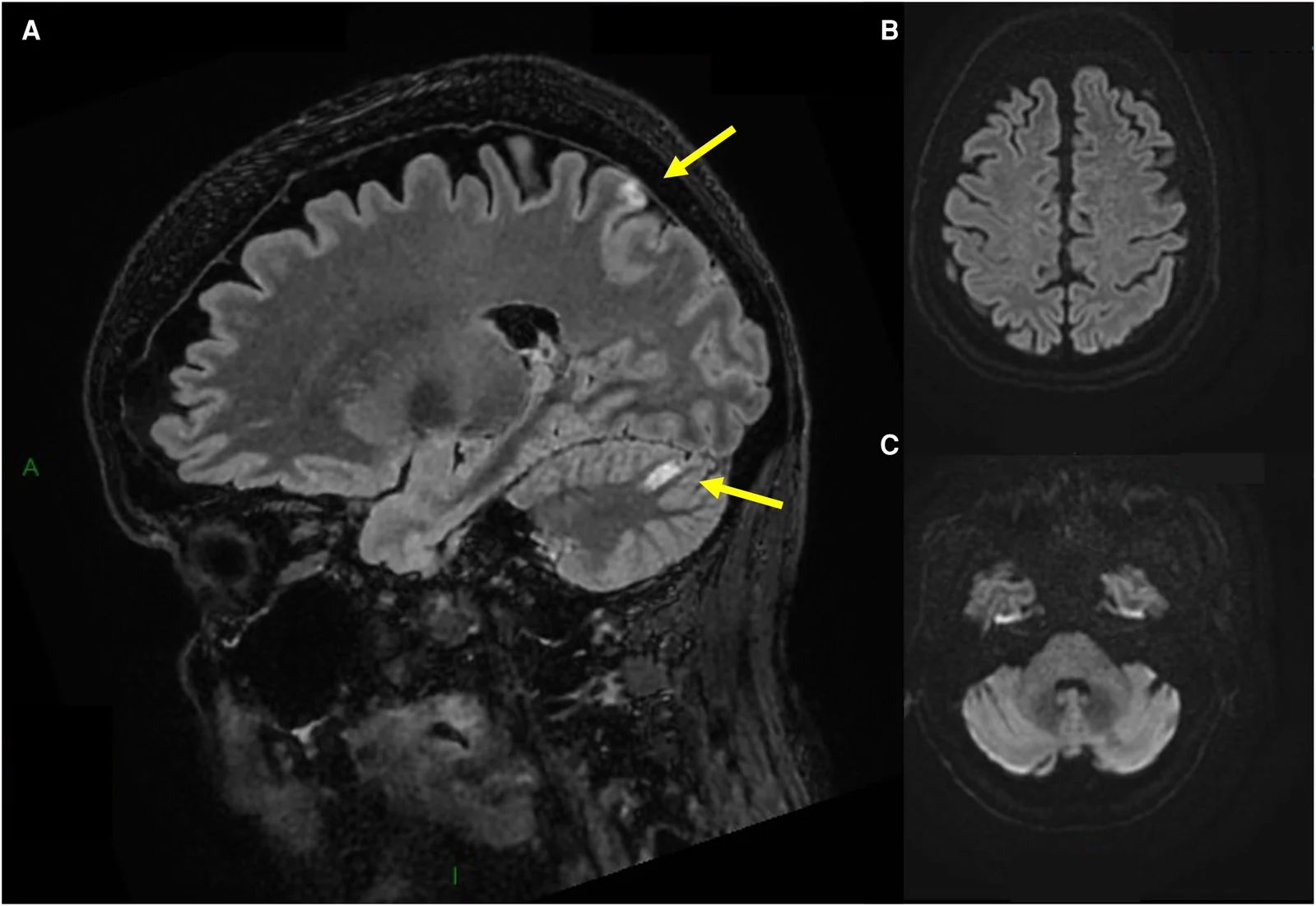
Brain magnetic resonance imaging performed after postoperative neurological deterioration. (*A*) Sagittal FLAIR sequence showing left cerebellar and left high parietal cortico-subcortical ischaemic sequelae. (*B*) Diffusion-weighted imaging at the same levels showing no evidence of recent ischaemia. (*C*) Corresponding axial image at the same levels showing no haemorrhagic transformation.

The subsequent course was further complicated by extensive deep venous thromboses and localized Candida albicans peritonitis, both successfully treated. The patient subsequently returned home without focal motor deficit and with mild residual attentional impairment. At 1-month follow-up, CT angiography confirmed persistent patency of the iliac-to-SMA bypass, although with reduced flow. At ∼6 months, CT angiography showed a stable aortic repair without endoleak or interval increase in aortic diameter compared with the 1-month examination. The reimplanted supra-aortic vessels remained patent without significant stenosis, and the native SMA remained patent despite mild narrowing where it traversed the stent struts. Conversely, the iliac-to-SMA bypass was nearly completely occluded, consistent with progressive competitive flow following restoration of antegrade perfusion through the native SMA. Colonoscopy was performed at 6 months, followed by elective restoration of intestinal continuity.

## Discussion

Acute type A aortic dissection complicated by mesenteric malperfusion is one of the most devastating presentations of acute aortic syndrome.^6,8^ Although mesenteric malperfusion occurs in only a minority of patients with ATAAD, it carries a severe prognostic burden and is consistently associated with markedly increased operative mortality. In this context, the usual imperative for immediate central aortic repair may conflict with the need to restore visceral perfusion before irreversible bowel injury and multiorgan failure develop. The present case illustrates a staged visceral-first strategy combining mesenteric revascularization, adjunctive endovascular treatment, sequential bowel surgery, and delayed total arch repair.

Current ESC, ACC/AHA, and EACTS/STS guidelines continue to endorse immediate central aortic repair as the standard treatment for ATAAD.^3–5^ However, they also acknowledge that, in selected patients with confirmed mesenteric malperfusion and established visceral ischaemia, initial visceral reperfusion before definitive aortic repair may be considered, although the supporting evidence remains limited. Once severe visceral ischaemia is established, cardiopulmonary bypass may exacerbate systemic inflammation, worsen coagulopathy, and precipitate irreversible multiorgan failure. The optimal sequence therefore remains debated, and patient selection appears central to decision-making.

The University of Michigan group reported favourable outcomes with upfront endovascular fenestration or stenting followed by delayed open repair, with progressive reductions in mortality over two decades of experience.^10,11^ Importantly, once arterial obstruction had been relieved, the risk of death from end-organ failure during the waiting period was reported to be far greater than the risk of aortic rupture.^11^ In their dedicated mesenteric malperfusion cohort, acute stroke, gross bowel necrosis at laparotomy, and serum lactate ≥6 mmol/L were independent predictors of death despite successful reperfusion,^9^ placing our patient, with an admission lactate of 8.2 mmol/L, in a particularly high-risk category. More recently, comparative data have suggested lower early mortality with reperfusion-first strategies, including SMA stenting, although these findings derive predominantly from observational studies and meta-analyses of non-randomized cohorts.^12,13^

These data do not, however, resolve the debate regarding the optimal therapeutic sequence. In a contemporary single-centre experience from the University of Pittsburgh, Brown *et al*. reported an in-hospital mortality of 20.6% in patients with visceral or iliofemoral malperfusion treated by immediate central aortic repair, arguing that prompt restoration of true lumen flow may reverse many cases of dynamic malperfusion before irreversible end-organ injury occurs.^14^ These differences likely reflect, at least in part, differences in patient selection rather than truly conflicting strategies. Emergency central repair remains the standard approach for ATAAD, while the optimal sequence in the presence of established mesenteric malperfusion syndrome remains uncertain. In highly selected patients with advanced bowel ischaemia, severe hyperlactataemia, or evolving multiorgan dysfunction, initial visceral reperfusion has been proposed as an alternative strategy, although evidence supporting this approach remains limited and largely observational.

In the present case, established mesenteric malperfusion and evolving haemodynamic instability prompted initial visceral reperfusion. Iliac-to-SMA bypass provided direct mesenteric revascularization while allowing simultaneous assessment of bowel viability,^15^ whereas TEVAR addressed the broader dynamic component of malperfusion. The latter entailed a risk of further aortic injury or rupture, mitigated by avoiding proximal oversizing and aggressive balloon expansion. Delaying central repair maintained exposure to catastrophic complications of the untreated proximal dissection, including rupture, tamponade, acute aortic regurgitation, coronary malperfusion, and dissection progression. Close monitoring may allow early detection but cannot eliminate these risks. The 6-day interval was not predetermined and reflected the time required to control visceral ischaemia, haemodynamic instability, and bleeding, with immediate central repair planned should any proximal complication occur.

This case highlights the importance of multidisciplinary coordination in the management of complex aortic emergencies, as decisions regarding initial visceral reperfusion, adjunctive endovascular treatment, repeated abdominal reassessment, and the timing of definitive aortic repair required close collaboration between cardiac, vascular, digestive, anaesthesia, intensive care, and interventional teams. Importantly, this case should not be interpreted as evidence of superiority of a visceral-first strategy or as supporting a change in current standard practice. Rather, it illustrates the individualized decision-making required in a highly selected patient with ATAAD and established severe mesenteric malperfusion syndrome, in whom the immediate risk of visceral ischaemia was weighed against the substantial risk of delaying central repair. Further multicentre data are needed to better define whether, and in which patients, such a staged approach may be appropriate.

## Supplementary Material

ytag693_Supplementary_Data

## Data Availability

The data underlying this article will be shared on reasonable request to the corresponding author.

## Lead author biography

Georges Abi Abdallah is an anaesthesiologist and intensivist at the European Hospital Georges Pompidou, Paris, and a researcher affiliated with Université Paris Cité and Inserm PARCC (UMR_S970). His clinical and research interests focus on acute aortic syndromes and extracorporeal cardiopulmonary resuscitation. He is actively involved in translational and clinical research on haemostatic disorders in critical illness, with a particular focus on acute aortic syndromes.

## Notes

**Consent:** The authors confirm that written consent for submission and publication of this case report, including images, supplementary material, and associated text, has been obtained from the patient, in line with COPE guidance.
